# Clinical and Pathogenetic Significance of Amylase Level and Microtomographic Index of Synovial Fluid in Various Joint Lesions

**DOI:** 10.17691/stm2022.14.6.05

**Published:** 2022-11-28

**Authors:** I.N. Schendrigin, L.D. Timchenko, I.V. Rzhepakovsky, S.S. Avanesyan, M.N. Sizonenko, W.-D. Grimm, S.N. Povetkin, S.I. Piskov

**Affiliations:** Associate Professor, Department of Intermediate Level Therapy; Stavropol State Medical University, 310 Mira St., Stavropol, 355017, Russia;; Professor, Chief Researcher, Interdepartmental Scientific and Educational Laboratory of Experimental Immunomorphology, Immunopathology, and Immunobiotechnology; North Caucasian Federal University, 1 Pushkin St., Stavropol, 355017, Russia;; Associate Professor, Leading Researcher, Interdepartmental Scientific and Educational Laboratory of Experimental Immunomorphology, Immunopathology, and Immunobiotechnology; North Caucasian Federal University, 1 Pushkin St., Stavropol, 355017, Russia;; Researcher, Interdepartmental Scientific and Educational Laboratory of Experimental Immunomorphology, Immunopathology, and Immunobiotechnology; North Caucasian Federal University, 1 Pushkin St., Stavropol, 355017, Russia;; Researcher, Interdepartmental Scientific and Educational Laboratory of Experimental Immunomorphology, Immunopathology, and Immunobiotechnology; North Caucasian Federal University, 1 Pushkin St., Stavropol, 355017, Russia;; Professor; Witten/Herdecke University, 50 A.-Herrhausen St., Witten, 58448, Germany; Training Master, Department of Physics and Technology of Nanostructures and Materials; North Caucasian Federal University, 1 Pushkin St., Stavropol, 355017, Russia;; Leading Researcher, Interdepartmental Scientific and Educational Laboratory of Experimental Immunomorphology, Immunopathology, and Immunobiotechnology; North Caucasian Federal University, 1 Pushkin St., Stavropol, 355017, Russia;

**Keywords:** joint pathology, synovial fluid, amylase activity, X-ray computed microtomography, X-ray density

## Abstract

**The aim of the investigation** was to study the level of amylolytic activity and microtomographic index of synovial fluid density as well as to substantiate their clinical and pathogenetic significance by identifying correlations with the known informative indicators reflecting characteristic features of the pathological process in various joint diseases.

**Materials and Methods:**

Samples of synovial fluid from 95 patients with various joint pathologies at the stage of the disease progression characterized by copious effusion into articular cavities have been examined. Synovial fluid samples obtained by knee arthrocentesis served as a material for the investigation. Conventional methods were used to determine the concentration of uric acid, inorganic phosphorus, total protein, and amylolytic activity level in the selected samples while X-ray density was identified by computed microtomography.

**Results:**

All samples of pathological joint fluid have shown a high level of amylolytic activity as compared to the synovial fluid from healthy joints. The relationship between the level of amylolytic activity in synovia and specific joint pathology has been identified. It has also been found that uric acid values, inorganic phosphorus concentrations, and total protein in various types of joint damage may influence X-ray density of the synovial fluid. Correlations between the studied indices have been established.

**Conclusion:**

New data on the level of synovia amylolytic activity has been obtained in one non-inflammatory and six different inflammatory diseases. Pathogenically determined correlation between the microtomographic index of synovial fluid density and concentrations of uric acid, inorganic phosphorus, total protein has been confirmed. Specific indicators of X-ray density of synovia in various joint pathologies as well as unidirectional and multidirectional data in comparison with the norm allow us to consider X-ray microtomography as a method that reveals additional details during investigation of synovial fluid density and brings new surrogate markers for the study of pathogenetic mechanisms of the development, differentiation, and treatment of various joint pathologies.

## Introduction

The main role in the pathogenesis of inflammatory degenerative diseases is given to synovial fluid (SF) and its qualitative parameters [1]. A liquid content in a healthy joint is represented, as a rule, by a true SF, the amount of which is extremely small and a composition is rather stable, whereas in the diseased joint, additional effusion is produced as an exudate (in inflammatory processes) or transudate (in degenerative lesions of non-inflammatory etiology) [2].

The quality of exudate and transudate and their volume are directly related to the permeability of the vascular wall and articular membranes. Fluid accumulation is often typical for the most severe stages of the pathological process irrespective of nosological types of articular pathology and causes more or less evident edema of the structural elements of the joint itself and periarthric tissues, which is a marked clinical sign of the lesion and often dictates the necessity of surgical manipulations.

There are reports [3, 4] demonstrating evidence of scientific and practical interest to specific aspects of qualitative indicators of the removed SF in joint lesions of various etiology. It is these indicators that allow one to judge about sophisticated pathogenetic mechanisms of articular pathologies and efficacy of their treatment and to predict the course of the disease. SF is the leading and sometimes the only factor for the proper diagnosis of joint diseases, since it is considered to be the most specific joint component connecting all its structural links and determining its morphofunctional state [5].

In the literature of the recent years, the results of mono- and complex researches reflecting clinical-morphological, immunological, and chemical SF compositions have been presented. For example, there appeared the works devoted to the study of immune cells [6], glucose level, activity of hyaluronidase, alkaline phosphatase, and lactate dehydrogenase in SF [7], proteome profile [8-10], and carbohydrate composition of synovia [11] in different articular pathologies. These data underlines to some extent a marked clinical-pathogenetic significance of almost each of the aforementioned indices. It is clearly seen how these indices reflect specific manifestations of the inflammatory response (alteration of the exudative processes as well): intensity of inflammatory fluid effusion, its biochemical and immunochemical properties, emigratory and proliferative cell reactions, and other characteristics.

In single reports, there are data on the presence and variations of the amylase level in the articular fluid in some joint lesions [5]. Since amylase is not synthesized in the joint, but is circulating in the bloodstream, its penetration in SF may be connected with the increased permeability in the vascular wall of the synovial membrane, which is a direct reflection of the exudation intensity. It is because of this phenomenon that amylolytic activity deserves attention as a promising parameter for prediction of exudative component specificity in dynamics.

Generally speaking, any information on the SF condition allows one to count on the effective control of inflammation intensity. There has been no unanimous recognition of different criteria priority in the qualitative SF analysis as yet. It is quite obvious that the greater number of the analyzed indices will help characterize the depth of the pathological process with greater statistical significance. However, investigations of SF should be conducted in the first 15 min of its delivery to the laboratory as longer storage causes biomaterial destruction [12]. In this connection, there arises a question on the search for the more informative and adequate integrative indicators for the assessment of the joint lesion degree. X-ray density may be such an indicator. There are examples of studying SF density using electric bioimpedance [13] and optical density [14].

X-ray computed microtomography, non-destructive imaging of the three-dimensional internal microstructure of an object by means of X-ray radiation, may be an alternative. This method is similar to computed tomography: a microfocus X-ray tube is illuminating the object while an X-ray chamber receives its magnified shadow projections. Based on thousands of projections obtained under various angles from the rotating object, the computer reconstructs the set of virtual object sections. The system transforms the results of scanning into realistic models and calculates internal morphometric parameters [15]. A high-degree resolution of microtomography can provide examination accuracy owing to the fixation of maximal number of the microstructural components of articular effusion.

**The aim of the investigation** was to study the level of amylolytic activity and microtomographic index of synovia density as well as to substantiate their clinical and pathogenic significance by identifying correlations with the traditional informative indicators (uric acid, inorganic phosphorus, total protein) reflecting characteristic features of the pathological process in various joint diseases.

## Materials and Methods

Samples of synovial fluid obtained by knee arthrocentesis from patients (n=95) treated in the Stavropol Regional Clinical Hospital (Russia) served as the material for investigations. These patients suffered from various joint pathologies at the stage of the disease progression characterized by copious effusion into articular cavities.

The groups of synovial fluid samples included rheumatoid arthritis (n=28), ankylosing spondyloarthritis (n=16), reactive arthritis (n=14), gouty arthritis (n=12), chronic synovitis (n=9), psoriatic arthritis (n=9), gonarthrosis (n=7). Samples (n=6) of SF from suddenly died people having no registered articular pathology were used as control samples.

The study complies with the Declaration of Helsinki (2013) and was approved by the Ethics Committee of the Stavropol Regional Clinical Hospital. Written informed consent for participation in the study and processing of personal data was obtained from all patients. All manipulations associated with collection, transportation, and investigation of the biomaterial were performed according to the appropriate recommendations [16, 17].

To analyze the content of phosphorus and uric acid in synovia, sample preparation was conducted. Synovial fluid (1 ml) was mixed with 10% solution of trichloracetic acid (1 ml) and centrifuged at 4000 rpm (MicroCL 17R centrifuge with cooling; Thermo Fisher Scientific, Great Britain).

Concentration of uric acid was determined with Folin’s reagent in the SF-102 spectrophotometer (IFF, Russia) at the 625-nm wavelength; inorganic phosphorus in the filtrate was measured by the molybdate method at wavelength of 630 nm.

The amount of total protein was assessed by the Warburg–Christian assay method with 100-fold dilution of the synovial fluid sample with subsequent spectrophotometry at the wavelengths of 260 and 280 nm.

The amylase level was tested using starch hydrolysis with enzymes of amylolytic complex up to dextrins with subsequent measurement of optical density of the iodine-starch complex on the SF-102 spectrophotometer.

X-ray density of freshly collected SF samples was studied using a Skyscan 1176 microtomograph (Bruker, Belgium). The samples were scanned in plastic test tubes at the following parameters: X-ray tube voltage — 45 kV; X-ray source current — 550 μA; Al filter — 0.2 mm; pixel size — 8.87 μm [18]. The scanning protocol included X-ray tube rotation of 180°; sample rotation at each step — 0.3; exposure time of 840 ms per image; frame averaging — 1. X-ray density of the synovia was calculated in Hounsfield units (HU) using CTAn v. 1.18.4.0 software (Bruker, Belgium) according to the protocol published in our previous work [19].

All quantitative parameters were determined using 3-fold analytical repetition. The acquired data were processed by Statistica 6.0 and Microsoft Excel 2010 software.

Normal distribution of the samples was checked using Shapiro–Wilk test. Taking into account that part of the data did not follow the law of normal distribution, non-parametric criteria were applied to assess statistical significance of differences between the samples. Kruskal–Wallis test was used as a preliminary statistical method with a subsequent pairwise comparison with the help of Mann–Whitney criterion. Differences were considered statistically significant at р≤0.006 (after recalculation by the number of comparisons). The results are represented as median (Me) and interquartile range (the 25^th^ and 75^th^ percentiles). Functional interrelations between the tested indicators were found using Spearman’s correlation analysis with indication of a 95% confidence interval (95% CI), statistical significance, and correlation coefficient (ρ).

## Results

The obtained data of the tested indicators in the normal SF were practically in agreement with the reference values given in the literature [20, 21].

A comparative analysis of concentrations of uric acid, phosphorus, and total protein in SF with the consideration of articular pathologies has revealed a number of differences. The indicator of synovial amylolytic activity also demonstrated a spread of values among the examined groups.

**Figure 1.**
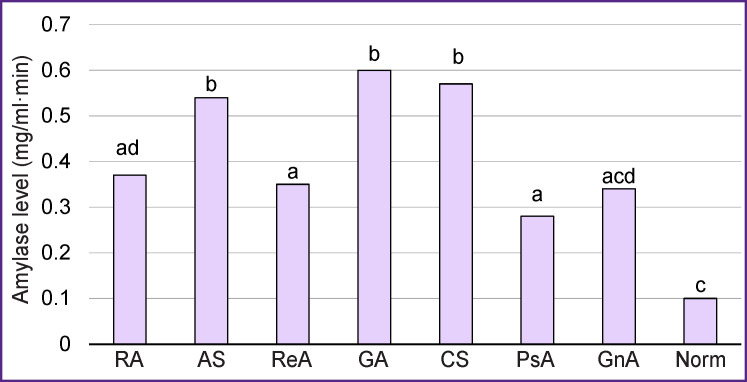
Median of amylolytic activity of synovial fluid in patients with various joint pathologies Here: RA — rheumatoid arthritis; AS — ankylosing spondyloarthritis; ReA — reactive arthritis; GA — gouty arthritis; CS — chronic synovitis; PsA — psoriatic arthritis; GnA — gonarthrosis. Values having no similar literal indices (a, b, or c) are statistically significantly different from each other (р≤0.006)

All samples of the pathological SF were characterized by relatively high values of amylolytic activity in comparison with the healthy joint samples. Besides, these values in the groups with ankylosing spondyloarthritis, gouty arthritis, and chronic synovitis were 5 times higher than the norm. In rheumatoid arthritis, reactive arthritis, psoriatic arthritis, and gonarthrosis, the level of amylolytic activity was almost three times higher than the normal value (Figure 1, the Table).

**Table:** Parameters of synovial fluid in patients with various joint pathologies, Me [Q1; Q3]

Diagnosis	Uric acid (mg/ml)	Inorganic (mg/phosphorus ml)	Total protein (%)	Amylolytic (mg/ml·activity min)	X-ray density (HU)
Rheumatoid arthritis (n=28)	0.065 [0.029; 0.112]^ad^	0.039 [0.037; 0.047]^a^	4.28 [3.80; 5.22]^a^	0.37 [0.25; 0.45]^ad^	48.58 [42.51; 51.69]^a^
Ankylosing spondyloarthritis (n=16)	0.055 [0.044; 0.075]^ad^	0.039 [0.035; 0.043]^a^	6.96 [5.95; 7.46]^c^	0.54 [0.50; 0.60]^b^	49.17 [36.88; 57.07]^ab^
Reactive arthritis (n=14)	0.060 [0.050; 0.11]^ad^	0.036 [0.027; 0.038]^a^	5.38 [4.48; 5.61]^ad^	0.35 [0.30; 0.36]^a^	47.07 [45.41; 53.75]^a^
Gouty arthritis (n=12)	0.100 [0.076; 0.118]^ab^	0.032 [0.030; 0.036]^ab^	4.05 [3.79; 6.28]^a^	0.60 [0.48; 0.69]^b^	42.52 [38.89; 50.71]^a^
Chronic synovitis (n=9)	0.090 [0.083; 0.110]^abd^	0.041 [0.030; 0.054]^a^	3.59 [3.36; 4.0]^a^	0.57 [0.47; 0.64]^b^	48.0 [43.52; 61.25]^ab^
Psoriatic arthritis (n=9)	0.026 [0.020; 0.039]^c^	0.037 [0.030; 0.041]^ab^	6.28 [5.72; 7.0]^c^	0.28 [0.25; 0.31]^а^	46.40 [46.0; 51.47]^ab^
Gonarthrosis (n=7)	0.065 [0.048; 0.075]^ad^	0.039 [0.032; 0.042]^a^	4.36 [4.04; 4.70]^ad^	0.34 [0.29; 0.35]^acd^	58.92 [55.88; 69.74]^b^
Norm (control) (n=6)	0.076 [0.066; 0.094]^ad^	0.021 [0.019; 0.024]^b^	0.024 [0.022; 0.034]^b^	0.10 [0.08; 0.12]^c^	62.23 [56.65; 69.24]^b^

Note. Values in one column having no similar literal indices (a, b, or c) are statistically significantly different from each other (р≤0.006).

Statistically significant positive correlation was established between the level of SF amylolytic activity and concentration of total protein in ankylosing spondyloarthritis (ρ=0.81; p=0.002; 95% CI: 0.46–0.94) and chronic synovitis (ρ=0.83; p=0.008; 95% CI: 0.30–0.95), and also the concentration of phosphorus in the sample of ankylosing spondyloarthritis (ρ=0.78; p=0.004; 95% CI: 0.40–0.93).

Interesting are first obtained comparative results of X-ray density for SF samples registered by computed microtomography in norm and in various joint lesions.

**Figure 2.**
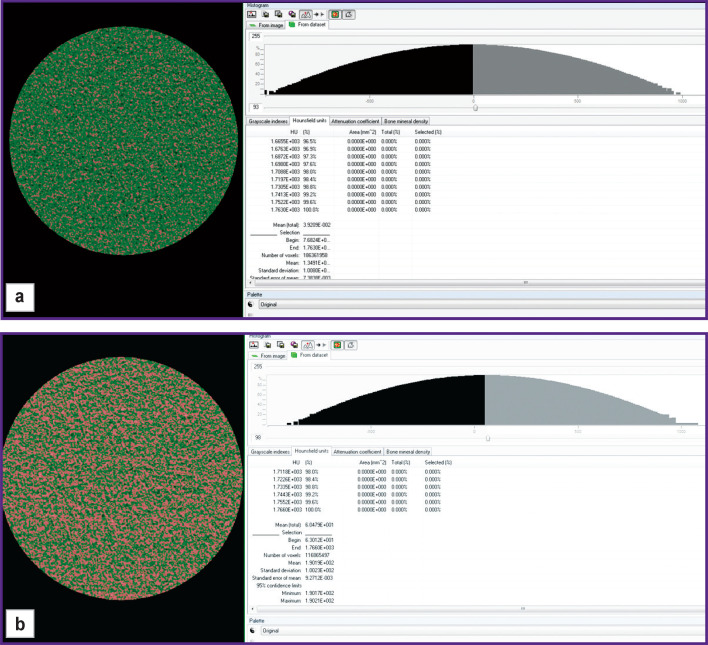
Determination of X-ray density using the CTAn software (v. 1.18.4.0; Bruker, Belgium): (a) water phantom; (b) normal synovia

The value of SF X-ray density for a healthy joint was 62.23 [56.65; 69.24] HU (Figure 2). In all samples of pathological synovial specimens except for the gonarthrosis group, this parameter appeared to be statistically significantly lower than that in SF of the healthy joint. The difference from normal values in the direction of reduction varied from 5.3 to 31.7% (see the Table).

**Figure 3.**
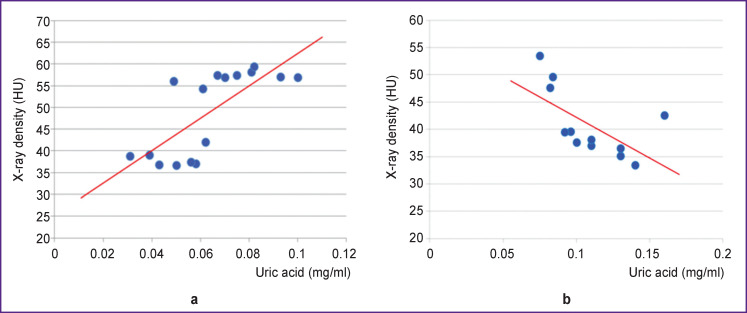
Interrelation of synovial fluid x-ray density with uric acid concentration: (a) ankylosing spondyloarthritis; (b) gouty arthritis

To establish the presence and degree of relationship between the mentioned pathogenetic and diagnostically significant characteristics of SF and X-ray density, correlation analysis was performed for each individual group of synovia samples. Correlation between X-ray density and uric acid level was only in ankylosing spondyloarthritis and gouty arthritis (Figure 3). And if in ankylosing spondyloarthritis the correlation was positive (ρ=0.69; p=0.001; 95% CI: 0.23–0.90), in gouty arthritis it bore a negative character (ρ=–0.62; p=0.012; 95% CI: 0.15–0.89).

**Figure 4.**
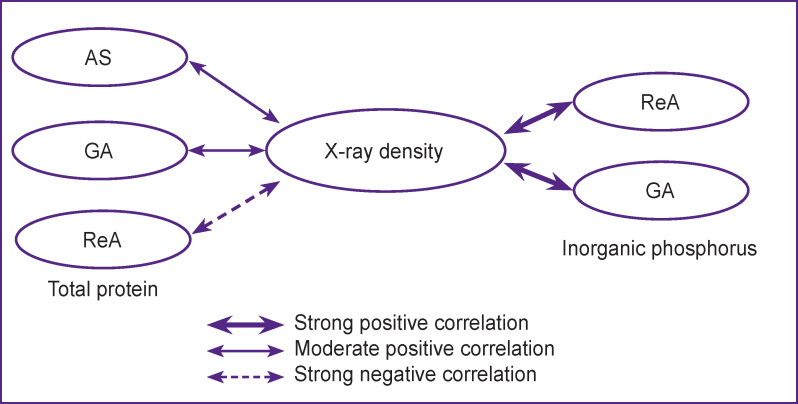
Correlation dependences between the value of X-ray density and concentrations of inorganic phosphorus and total protein in synovia in various joint pathologies Here: AS — ankylosing spondyloarthritis; GA — gouty arthritis; ReA — reactive arthritis

The groups with reactive arthritis (ρ=0.74; p=0.043; 95% CI: 0.27–0.92) and gouty arthritis (ρ=0.87; p=0.021; 95% CI: 0.28–0.95) demonstrated a strong positive correlation between X-ray density and amount of inorganic phosphorus in SF (Figure 4).

A moderate positive correlation between the content of total protein and X-ray density of SF was observed in ankylosing spondyloarthritis (ρ=0.60; p=0.032; 95% CI: 0.11– 0.86) and gouty arthritis (ρ=0.55; p=0.041; 95% CI: 0.10–0.86). The reactive arthritis sample, on the contrary, was characterized by a strong negative correlation (ρ=–0.89; p=0.024; 95% CI: 0.62–0.97) (see Figure 4).

A moderate positive correlation was established between X-ray density and SF amylolytic activity in the groups with rheumatoid arthritis (ρ=0.63; p=0.017; 95% CI: 0.30–0.82) and ankylosing spondyloarthritis (ρ=0.50; p=0.04; 95% CI: 0.20–0.81).

## Discussion

In the present study, in order to establish clinical-pathogenetic and diagnostic significance of the amylase level and microtomographic radiodensity of SF, emphasis was laid on correlations of these characteristics with three indices (uric acid, inorganic phosphorus, total protein), dynamics of which in the development and manifestation of the joint diseases with various etiopathogenesis does not raise any doubts. The choice of these indicators was determined owing not only to their high informativity for clinical practice and relatively available determination methods [22] but also the previously established differences in the degree of X-ray density [23, 24].

Uric acid acts as a physiological regulator of inflammation caused by tissue damage [25] and is a marker of severity and progression of joint tissue lesion [26]. The amount of inorganic phosphorus is associated with the level of cell proliferation and their exchange in the cartilage and serves as a marker of structural outcome in articular pathology [27]. Total protein concentration in SF characterizes permeability of hemosynovial barrier and reflects the level of synovial plasma flow, since protein is delivered into synovia by blood transudate [28]. This parameter reflects quantitatively the severity of microvascular joint lesions [29].

The increase of uric acid concentration in the synovial specimens in patients with gouty arthritis, noted by us, is quite logic [30] and is in line with the results of Vaidya et al. [31], who prove that this indicator may be used as an alternative test to polarized microscopy not always available for gout diagnosis.

Concentration of uric acid in blood serum in psoriatic arthritis does not differ from the norm or tends to increase [32, 33]. In the present study, relatively low values of the uric acid level were found in the SF specimens of the patients with this pathology, which agrees with the results obtained by Teplova et al. [34], according to which uric acid is not always detected in SF despite the increase of its level in the blood.

Relatively low values of the uric acid level were also observed in the specimens of synovia from patients with ankylosing spondyloarthritis. Some specialists [35] believe that this fact confirms the value of the given indicator in differentiating ankylosing spodyloarthritis from gouty arthritis having similar clinical picture.

Concentration of phosphorus in all SF samples was within the reference values [21] and did not have statistically significant intergroup differences in pathologies. Besides, they were higher in some groups than in the normal SF specimens, which runs counter to the literature data, in particular regarding gonarthrosis [1]. At the same time, Zar et al. [36] point to the considerable increase of the amount of phosphorus ions in SF accompanying, along with calcium ions, activation of the mechanisms of protective biominiralization which is directed to neutralization of the toxic elements destroying cartilaginous tissue. This confirms the interest to investigation of inorganic phosphorus level in SF and its role in the development of non-inflammatory joint pathology.

All samples of pathological SF were characterized by a higher concentration of total protein in comparison with the control specimens of synovia. This supplements the recent results of clinical investigations of osteoarthrosis and arthritic changes [1], showing an elevated concentration of total protein in the articular liquor. The greatest amount of protein was noted in the synovial specimens of patients with ankylosing spondyloarthritis and psoriatic arthritis; minimal amount was found in chronic synovitis. The tendency to increase of protein in SF above the norm was observed even in gonarthrosis which is a pathology of non-inflammatory etiology, which may indicate a marked significance of the protein in formation and manifestation of different mechanisms of joint lesion characterizing not only phenomenon of inflammation but non-inflammatory structural degradations of the joint tissues (impairment of vessel integrity and synovial membrane), immune and enzymatic processes accompanied by generation and biotransformation of SF protein molecules.

According to the study performed by Synyachenko [5], the highest level of amylase is noted in SF of patients with rheumatoid arthritis. However, according to our data, the first place was shared by the samples of synovia in ankylosing spondyloarthritis, gouty arthritis, and chronic synovitis, which may designate the intensity of inflammation determined by the degree of vessel damage. This is logically confirmed by the positive correlation, registered by us, between the level of amylolytic activity and protein concentration in SF of samples with ankylosing spondyloarthritis and chronic synovitis.

Taking into consideration amylolytic activity in SF of a healthy joint, as well as the fact that amylase is not synthesized in the joint but is circulating in the bloodstream, its increase in SF is likely to be caused either by the elevation in permeability of the vascular wall of synovial membrane (which is a direct reflection of exudation intensity) or is connected with conditions accompanied by the amylase activity increase in the blood. For example, macroamylasemia is often associated with rheumatoid arthritis [37].

The obtained result of correlation analysis between X-ray density and the uric acid level in the group of gouty arthritis logically coincides with the above-mentioned maximal amount of uric acid and correlates with the data presented by Mironov et al. [23] on radiolucency of uric acid salts.

The established differences of correlations between the total protein content and X-ray density of SF in the experimental groups are likely to be associated with simple concentration of proteins in SF and also with conformational changes of the protein molecules or accumulation of proteolysis products — low and medium molecular organic compounds in the synovia due to a disbalance of the mechanisms of the proteinase– inhibitors system which is often accompanies arthritis development [38].

Undoubtedly, other components concentrated in SF, for example, lipids may also influence its X-ray density. Lipid substances are known to have negative X-ray density relative to water and blood [39]. SF lipid profiles are significantly different in healthy people and patients with joint pathology [40]. The amount and spectrum of lipid substances in SF may vary depending on the character of joint lesion. For example, according to Oliviero et al. [41], concentration of lipids in SF is higher in patients with arthritides than in those with arthroses. This also agrees with our results, according to which the values of X-ray density of the SF samples from patients with arthritides (rheumatoid arthritis, ankylosing spondyloarthritis, reactive arthritis, gouty arthritis, psoriatic arthritis) were lower than those for the SF specimens in gonarthrosis.

In general, analysis of the literature data and the results of the present study allow us to conclude that qualitative indicators of SF reflect the character and intensity of the pathological process in a joint.

We propose to refer the level of amylolytic activity and microtomographic index of synovial fluid density to the number of significant characteristics of SF (with undefined pathogenetic role).

### Study limitations

Since it was not our task to study the considered parameters in the present work taking into account other important factors, for example, stages of the pathological process and/or microbial contamination, further investigations are necessary to confirm pathogenetic and diagnostic informativity of the amylolytic activity level and microtomographic index of synovia fluid density in the monitoring of joint lesion progression and therapy efficacy. It should be also taken into consideration that samples of separate groups of SF specimens in our study were rather small. In this connection, the expediency of widening the spectrum of investigation of these indicators on a substantially greater number of patients aiming at concretization of the hypothesis proposed by us concerning the differential and diagnostic significance of these parameters in diverse types of joint pathology.

## Conclusion

New data on the level of synovia amylolytic activity obtained by us in one non-inflammatory and six different inflammatory diseases show the elevation of this indicator in all pathological processes in comparison with the norm. At the same time, differences of the amylolytic activity level between nosological groups of inflammatory diseases have been noted.

Pathogenetically determined correlation of various degrees between microtomographic indicator of synovial fluid density and concentration of uric acid, inorganic phosphorus, and total protein has been validated.

Specific values of synovial fluid X-ray density in different joint pathologies in comparison with the norm allow one to consider X-ray microtomography as a method disclosing additional details in the process of investigation of synovial fluid density and bringing new surrogate markers for the study of pathogenetic mechanisms of the development, differentiation, and treatment of various joint pathologies.

## References

[1] Ryabinin S.V., Peleshenko E.I., Ryabinina E.I., Samodai V.G. (2020). Examination of the level of certain physicochemical indices of synovial fluid in normal and during gonartrosis.. Prikladnye informacionnye aspekty mediciny.

[2] Kotelkina A.A., Struchko G.Yu., Merkulova L.M., Kostrova O.Yu., Stomenskaya I.S., Timofeeva N.Yu. (2017). Characteristics of synovial fluid under normal conditions and in some patthological processes.. Acta Medica Eurasica.

[3] Ingale D., Kulkarni P., Electricwala A., Moghe A., Kamyab S., Jagtap S., Martson A., Koks S., Harsulkar A. (2021). Synovium-synovial fluid axis in osteoarthritis pathology: a key regulator of the cartilage degradation process.. Genes (Basel).

[4] Mustonen A.M., Käkelä R., Joukainen A., Lehenkari P., Jaroma A., Kääriäinen T., Kröger H., Paakkonen T., Sihvo S.P., Nieminen P. (2021). Synovial fluid fatty acid profiles are differently altered by inflammatory joint pathologies in the shoulder and knee joints.. Biology (Basel).

[5] Synyachenko O.V. (2008). Modern aspects synovyal liquid’s analysis.. Ukrains'kij revmatologicnij zurnal.

[6] Kriegova E., Manukyan G., Mikulkova Z., Gabcova G., Kudelka M., Gajdos P., Gallo J. (2018). Gender-related differences observed among immune cells in synovial fluid in knee osteoarthritis.. Osteoarthritis Cartilage.

[7] Hammodat Z.M., Mustafa L.A. Biochemical studies on synovial fluid, serum from rheumatoid arthritis patients. (2018). Raf J Sci.

[8] Birkelund S., Bennike T.B., Kastaniegaard K., Lausen M., Poulsen T.B.G., Kragstrup T.W., Deleuran B.W., Christiansen G., Stensballe A. (2020). Proteomic analysis of synovial fluid from rheumatic arthritis and spondyloarthritis patients.. Clin Proteomics.

[9] Timur U.T., Jahr H., Anderson J., Green D.C., Emans P.J., Smagul A., van Rhijn L.W., Peffers M.J., Welting T.J.M. (2020). Identification of tissue-dependent proteins in knee OA synovial fluid.. Osteoarthritis Cartilage.

[10] Ali N., Turkiewicz A., Hughes V., Folkesson E., Tjörnstand J., Neuman P., Önnerfjord P., Englund M. (2022). Proteomics profiling of human synovial fluid suggests increased protein interplay in early-osteoarthritis (OA) that is lost in late-stage OA.. Mol Cell Proteomics.

[11] Berthoud O., Coiffier G., Albert J.D., Gougeon-Jolivet A., Goussault C., Bendavid C., Guggenbuhl P. (2020). Performance of a new rapid diagnostic test the lactate/ glucose ratio of synovial fluid for the diagnosis of septic arthritis.. Joint Bone Spine.

[12] Jaggard M.K.J., Boulangé C.L., Graça G., Akhbari P., Vaghela U., Bhattacharya R., Williams H.R.T., Lindon J.C., Gupte C.M. (2021). The influence of sample collection, handling and low temperature storage upon NMR metabolic profiling analysis in human synovial fluid.. J Pharm Biomed Anal.

[13] Krishnan G.H., Nanda A., Natarajan A.R. (2015). Synovial fluid density measurement for diagnosis of arthritis.. Biomed Pharmacol J.

[14] Seagal Z.M., Surnina O.V., Brindin V.V., Seagal S.Z. (2018). Development of intraorganic transylumination and ultrasound monitoring in rheumatoid arthritis.. Dnevnik kazanskoj medicinskoj skoly.

[15] Orhan K. (2020). Micro-computed tomography (micro-CT) in medicine and engineering..

[16] Sikilinda V.D., Alabut A.V. (2018). Protocols of technique of punctions of joints and treatment blocades in trauma and orthopedic diseases of support-moving apparatus.. Glavnyj vrac Uga Rossii.

[17] Prikaz Minzdrava Rossii ot 12.11.2012 No.900n “Ob utverzhdenii Poryadka okazaniya meditsinskoy pomoshchi vzroslomu naseleniyu po profilyu “revmatologiya”.

[18] Rzhepakovsky I., Siddiqui S.A., Avanesyan S., Benlidayi M., Dhingra K., Dolgalev A., Enukashvily N., Fritsch T., Heinz V., Kochergin S., Nagdalian A., Sizonenko M., Timchenko L., Vukovic M., Piskov S., Grimm W.D. (2021). Anti-arthritic effect of chicken embryo tissue hydrolyzate against adjuvant arthritis in rats (X-ray microtomographic and histopathological analysis).. Food Sci Nutr.

[19] Nagdalian A.A., Rzhepakovsky I.V., Siddiqui S.A., Piskov S.I., Oboturova N.P., Timchenko L.D., Lodygin A.D., Blinov A.V., Ibrahim S.A. (2021). Analysis of the content of mechanically separated poultry meat in sausage using computing microtomography.. J Food Compos Anal.

[20] Matveeva E.L., Spirkina E.S., Gasanova A.G. Biochemical composition synovial fluid knee normal people. (2015). Uspehi sovremennogo estestvoznania.

[21] Slack S.M. (2020). Properties of biological fluids.. Biomaterials science (4th edition)..

[22] Matveeva E.L., Gasanova A.G., Spirkina E.S. (2012). Prospects of synovial fluid investigation for clinical practice (review of literature).. Genij ortopedii.

[23] Mironov M.P., Zavadovskaya V.D., Zorkaltsev M.A., Kurazhov A.P., Fomina S.V., Shulga O.S., Zhogina T.V., Perova T.B. (2021). The possibility of using radiology modalities in the diagnosis of crystalline arthropathy.. Bulleten' sibirskoj mediciny.

[24] Sudhyadhom A. (2020). On the molecular relationship between Hounsfield unit (HU), mass density, and electron density in computed tomography (CT).. PLoS One.

[25] Kono H., Chen C.J., Ontiveros F., Rock K.L. (2010). Uric acid promotes an acute inflammatory response to sterile cell death in mice.. J Clin Invest.

[26] Denoble A.E., Huffman K.M., Stabler T.V., Kelly S.J., Hershfield M.S., McDaniel G.E., Coleman R.E., Kraus V.B. (2021). Uric acid is a danger signal of increasing risk for osteoarthritis through inflammasome activation.. Proc Natl Acad Sci U S A.

[27] Doherty M., Belcher C., Regan M., Jones A., Ledingham J. (1996). Association between synovial fluid levels of inorganic pyrophosphate and short term radiographic outcome of knee osteoarthritis.. Ann Rheum Dis.

[28] Spirkina E.S., Matveeva E.L., Gasanova A.G. (2013). Comparative characteristics of biochemical composition of synovial fluid of knee and elbow human joints.. Bulleten' Vostocno-Sibirskogo naucnogo centra Sibirskogo otdelenia Rossijskoj akademii medicinskih nauk.

[29] Güler N., Uçkan S., Imirzalıoğlu P., Açıkgözoğlu S. (2005). Temporomandibular joint internal derangement: relationship between joint pain and MR grading of effusion and total protein concentration in the joint fluid.. Dentomaxillofac Radiol.

[30] Lipatov I.A., Buksha I.A. (2021). Pathochemical processes in gout.. Vestnik Celabinskogo gosudarstvennogo universiteta. Obrazovanie i zdravoohranenie.

[31] Vaidya B., Bhochhibhoya M., Nakarmi S. (2018). Synovial fluid uric acid level aids diagnosis of gout.. Biomed Rep.

[32] Yanysheva A.V. (2009). Metabolic disturbances in psoriatic arthritis.. Sibirskij medicinskij zurnal.

[33] Koroy P.V. (2016). Psoriatic arthritis.. Vestnik molodogo ucenogo.

[34] Teplova L.V., Eremeeva A.V., Baykova O.A., Suvorova N.A. (2017). Rheumatic manifestations of hypothyroidism.. Sovremennaa revmatologia.

[35] Barskova V.G., Kudaeva F.M. (2005). Differential diagnosis of gouty arthritis.. Consilium Medicum.

[36] Zar V.V., Voloshin V.P., Shatochina S.N., Petushkova L.Yu., Shabalin V.N. (2012). Morphologic structures of synovial fluid in diagnosis of osteoarthrosis: condition and perspectives.. Al’manah kliniceskoj mediciny.

[37] Koltunov A.S., Alekseenko S.A., Koltunov S.S. (2019). A case report of macroamylasemia.. Dal'nevostocnyj medicinskij zurnal.

[38] Ivanova S.V. (2015). Parameters of the proteolytic system of the synovial fluid as diagnostic markers of certain forms of arthritis.. Vestnik Vitebskogo gosudarstvennogo medicinskogo universiteta.

[39] Hofer M., Kut’ko A.P., Pleshkova F.I., Ipatova V.V., Kut’ko A.P., Pleshkov F.I., Ipatov V.V., Trufanov G.E. (2008). Pod red. Trufanova G.E. [Computed tomography. Basic guide.. Komp’yuternaya tomografiya. Bazovoe rukovodstvo. Per. s angl..

[40] Zhang K., Ji Y., Dai H., Khan A.A., Zhou Y., Chen R., Gui J. (2021). High-density lipoprotein cholesterol and apolipoprotein A1 in synovial fluid: potential predictors of disease severity of primary knee osteoarthritis.. Cartilage.

[41] Oliviero F., Lo Nigro A., Bernardi D., Giunco S., Baldo G., Scanu A., Sfriso P., Ramonda R., Plebani M., Punzi L. (2012). A comparative study of serum and synovial fluid lipoprotein levels in patients with various arthritides.. Clin Chim Acta.

